# Protective Effects of Inulin on Stress-Recurrent Inflammatory Bowel Disease

**DOI:** 10.3390/ijms25052494

**Published:** 2024-02-21

**Authors:** Yao Du, Kanta Kusama, Koki Hama, Xinyue Chen, Yu Tahara, Susumu Kajiwara, Shigenobu Shibata, Kanami Orihara

**Affiliations:** 1 School of Life Science and Technology, Tokyo Institute of Technology, Yokohama 226-8501, Japan; du.y.ac@m.titech.ac.jp (Y.D.); chen.x.ac@m.titech.ac.jp (X.C.); kajiwara.s.aa@m.titech.ac.jp (S.K.); 2 Laboratory of Physiology and Pharmacology, School of Advanced Science and Engineering, Waseda University, Tokyo 162-8480, Japan; swmsoc12bskft18@akane.waseda.jp (K.K.); koby_frekoi418@ruri.waseda.jp (K.H.); yutahara@hiroshima-u.ac.jp (Y.T.); shibatas@waseda.jp (S.S.); 3 Graduate School of Biomedical and Health Sciences, Hiroshima University, Hiroshima 734-8553, Japan

**Keywords:** inulin, IBD, stress, SCFAs, cytokines, mucin, ER stress

## Abstract

Inflammatory bowel disease (IBD) is a chronic inflammatory condition of the digestive tract and is closely associated with the homeostasis of the gut microbiota. Inulin, as a natural prebiotic, displays anti-inflammatory activity and maintains equilibrium of the intestinal microbiota. In this study, our research aimed to explore the potential of inulin in enhancing intestinal immunity and reducing inflammation in stress-recurrent IBD. In this study, a co-culture intestinal epithelium model and a stress-recurrent IBD mouse model was used to examine the protective effects of inulin. It was observed that inulin digesta significantly reduced pro-inflammatory cytokine expression (*CXCL8/IL8* and *TNFA*) and increased *MUC2* expression in intestinal epithelial cells. In vivo, our findings showed that Inulin intake significantly prevented IBD symptoms. This was substantiated by a decrease in serum inflammatory markers (IL-6, CALP) and a downregulation of inflammatory cytokine (*Il6*) in colon samples. Additionally, inulin intake led to an increase in short-chain fatty acids (SCFAs) in cecal contents and a reduction in the expression of endoplasmic reticulum (ER) stress markers (CHOP, BiP). Our results highlight that inulin can improve stress-recurrent IBD symptoms by modulating microbiota composition, reducing inflammation, and alleviating ER stress. These findings suggested the therapeutic potential of inulin as a dietary intervention for ameliorating stress-recurrent IBD.

## 1. Introduction

Inflammatory bowel disease (IBD) is a chronic inflammatory disease in the digestive tract, according to the condition, it can be mainly divided into Ulcerative colitis (UC) and Crohn’s disease (CD). As for the etiology, environmental factors, genetic factors, and the disturbance of the balance of the immune response reaction between the intestinal microflora and the large intestine mucosa are considered, however the concrete factor is not clarified [1,2]. IBD patients are more than 0.4% of the world’s population which mainly distributed in Europe and North America and is expected to increase over time. Researchers believed that Western pattern diet (high fat, high flesh, low fruit, low vegetable) has led to a higher risk of IBD in Europa and America [3]. This may be related to immune dysregulation caused by obesity [4]. Patients with IBD are known to have a high risk of developing colorectal cancer, and as the age continues, the risk of developing cancer is inevitable.

Stress has been reported to be important in the recurrence of IBD. Stress is known to alter animal behavior and homeostasis by regulating nerve, endocrine and immune functions. Biological responses to stress include both hypothalamic pituitary adrenal (HPA) axis and activation of the sympathetic adrenal medulla system. Stress causes glucocorticoids and catecholamines to be released from the adrenal and sympathetic nerve terminals and in the cortex of adrenal medulla [5]. Some paper showed that acute stress decreased the level of IgA in the gut and reduces the cytoplasm of the major inducible sites of IgA production, which is caused by the migration of lymphocytes in part [6,7]. Increase in endogenous glucocorticoid by restraint stress reduces the number of Peyer cell lines through a mechanism associated with apoptotic cell death [8,9]. All this shows that excessive stress reduces intestinal immunity and has various effects on the body.

Prebiotics, as an important nutrition source of probiotics, are consist with selectively fermented short-chain carbohydrates that are not degraded or absorbed in the upper portion of the digestive tract and show the benefits on health by promoting probiotics growth and improving intestinal flora balance [10]. Prebiotic intake stimulates IgA, immunomodulatory interleukin and reduces inflammatory interleukin which regulates intestinal immunity [11]. Prebiotic intake also promotes the production of short chain fatty acids (SCFA) and lowers cecal pH. This prevents colonization by acid-sensitive enteric pathogens and specifically increases beneficial enterobacteria within the host intestinal tract. In addition, prebiotics also have many functions such as promoting mucosal cell differentiation and epithelial barrier function, regulating cell apoptosis, growth arrest and cell differentiation, and regulating glutathione S-transferase and histone acetylation patterns [12,13]. Therefore, prebiotics can improve intestinal immunity and maintaining intestinal environment. However, no evidence showed that the effects of these benefits on the IBD which induced by stress.

Inulin is a natural polydisperse carbohydrate extracted from chicory. Inulin belongs to fructose containing polymers, also known as fructans, which made up of linear fructose units tied by a β-(2-6) glycosidic bond [14]. Previous studies have reported that inulin as water soluble storage polysaccharide have nutritional and health benefits. Therefore, in this study inulin was used to examine the effect of prebiotics on reduced immunity induced by stress. In order to aim specifically to investigate the preventing/protecting effect of inulin on the recurrence of IBD induced by stress loading, the timing of inulin treatment was adjusted and examined.

## 2. Results

### 2.1. The Effect of Inulin Digesta on Immunity Reactions in Intestinal Epithelial Cells

**Figure 1:**
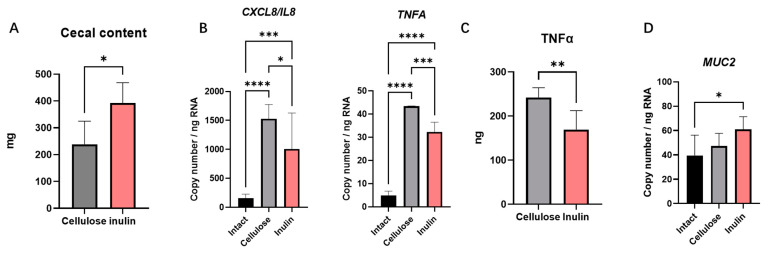
1 week inulin intake increased mice cecal content weight (**A**). Inulin digesta extracted from cecal content downregulated the mRNA expression level of *CXCL8/IL8* and *TNFA* (**B**). Inulin digesta also reduced TNFα released from cells (**C**). Inulin digesta increased the mRNA expression level of *MUC2* (**D**). Data are expressed as mean ± SEM values. N = 3 at each group and experiment was duplicate. A representative data from two independent trial were shown. * *p* < 0.05, ** *p* < 0.01, *** *p* < 0.001, **** *p* < 0.0001, unpaired *t*-test and one-way ANOVA between Intact, Inulin and Cellulose.

The effects of inulin intake were investigated on intestinal inflammation. As it is known, the metabolite of intestinal microbiota plays an important role in the intestinal immune system. Therefore, in this study an intestinal epithelial co-culture model was established with Caco-2 cells and HT-29 cells and treated with cecal digesta to confirm the anti-inflammatory effects of inulin. Cellulose, a homopolymer of glucose, was utilized as the positive control group be-cause the dietary fiber contained in normal diet is cellulose [15]. In this study, the cecal content weight change also suggested that inulin changed the intestinal flora (Figure 1A) [16].

In this study CXCL8/IL8 and TNFA were selected as target proinflammation cytokines to assess the level of inflammation in this study. Real-time PCR data showed a significant increase of *CXCL8/IL8* and *TNFA* mRNA expression in the cellulose group after 2 h treatment compared to the intact group (Figure 1B). This result suggested the presence of pro-inflammatory factors in cecal content. In contrast, the mRNA expression of *CXCL8/IL8* and *TNFA* in the inulin group was significantly reduced compared to the cellulose group (Figure 1B). This substantiated inulin intake may inhibit the pro-inflammatory effect of cecal content. To prove this view, ELISA analysis was done to measure the levels of the pro-inflammatory factor TNFα in the supernatant. As expected, the TNFα concentration in the supernatant of the inulin group was significantly lower than cellulose group (Figure 1C). This confirms the ability of inulin intake to inhibit intestinal inflammatory responses. Additionally, the mRNA expression of *MUC2* showed a significant increase in the inulin group compared to the intact group (Figure 1D).

### 2.2. The Effect of the Inulin Intake on Inflammation in Stress Model

**Figure 2:**
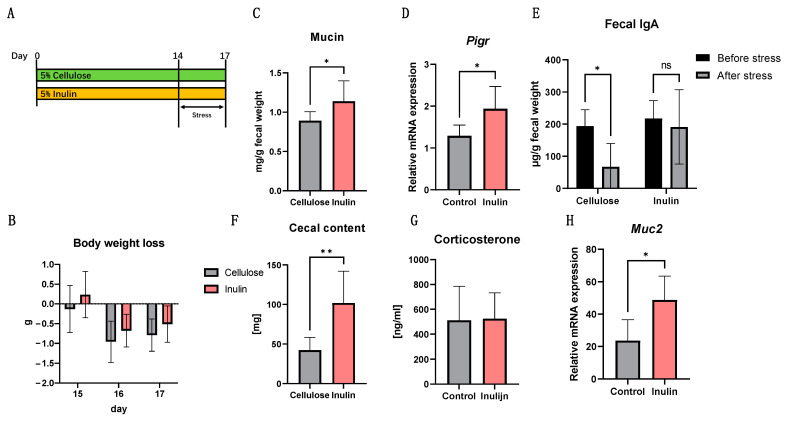
Inflammation was induced by acute stress in ICR (8 weeks old, N = 8/group) mice. Mice were fed with cellulose (5%) or inulin (5%) for 2 weeks. Acute stress was performed from day 14 to day 17 (**A**). 4 days stress treatment induced mice body weight loss (**B**). Cecal content weight (**C**) and serum corticosterone concentrations (**D**) after stress. Inulin treatment prevented the reduction of fecal IgA (**E**) and mucin (**F**). mRNA expression level of *Pigr* (**G**) and *Muc2* (**H**) in colon was improved. Data are expressed as mean ± SEM values. N = 6–8 at each group and experiment was duplicate. * *p* < 0.05, ** *p* < 0.01, unpaired *t*-test between Inulin and Cellulose.

In the next experiment, the impact of inulin intake on the intestinal immunity of mice stress model was investigated. The experiment involved two groups: the cellulose group and the inulin group. The cellulose group had a diet containing 5% cellulose for two weeks, while the inulin group had a diet containing 5% inulin, both freely fed. On the final day of the special diet period at ZT2, feces and blood were collected, as the pre-group. Subsequently, restraint stress was applied at ZT0-2 for 3 days. On the final day of the stress period at ZT2, feces and blood were collected before dissection (Figure 2A). Results showed that body weight decreased during the restraint stress period (Figure 2B). Additionally, corticosterone concentration in serum significantly increased in both groups after restraint stress (Figure 2D). This proved that a 3-days restraint stress can exert adverse effects in mice. While the serum corticosterone concentration in the inulin group did not exhibit significant alterations, the weight loss was less than that observed in the cellulose group (Figure 2C).

The restraint stress downregulated the concentration of IgA in the stools of the cellulose group, which are indicative of intestinal immune activity (Figure 2E). Conversely, inulin intake showed no significant change in the concentration of IgA and increased mucin concentration after regulated restraint stress compared with cellulose (Figure 2E,F). Consistent with these findings, inulin group mRNA expression levels in mice colonic samples demonstrated a significant upregulation in the mRNA expression of *Pigr* and *Muc2* compared with cellulose group (Figure 2G,H). 

### 2.3. The Effect of Inulin on IBD Symptoms in Stress-Recurrent IBD Model

**Figure 3:**
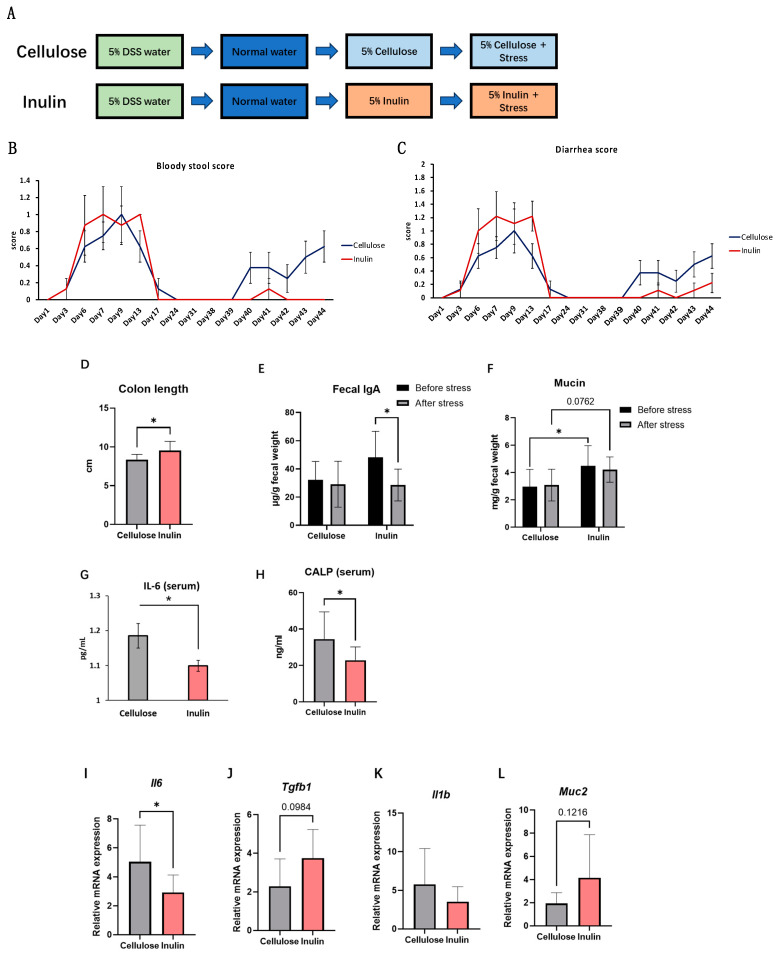
Inflammatory bowel disease (IBD) was induced by 2 weeks 5% DSS water feeding in ICR (8 weeks old, N = 8/group) mice. After 2 weeks recovery, mice were fed with cellulose (5%) or inulin (5%) for 2 weeks. Acute stress was performed for two weeks for recurrence of IBD through restraint stress (**A**). Bloody stool score (**B**), diarrhea score (**C**) and colon length (**D**) showed the level of IBD. Fecal IgA concentration (**E**) and fecal mucin concentration (**F**) were measured before and after stress. Inulin treatment downregulated serum IL-6 (**G**) and CALP (**H**). mRNA expression level of *Il6* (**I**), *Tgfb1* (**J**), *Il1b* (**K**) and *Muc2* (**L**) in colon was improved. Data are expressed as mean ± SEM values. N = 6–8 at each group and experiment was duplicate. * *p* < 0.05, unpaired *t*-test and two-way ANOVA between Inulin and Cellulose.

In the next experiment, whether inulin could reduce IBD symptoms in a mouse model that shows recurrence by stress exposure was examined. The downregulated intestinal immune system induced by stress is a potential reason for the recurrence of IBD [17]. Consequently, referencing a previous study [17], this study established a mice model of recurrence IBD which using dextran sulfate sodium (DSS) induced IBD symptoms first and led to the recurrence of intestinal inflammation through restraint stress after a period of recovery (Figure 3A). In this model, the recovered mice exhibited a colonic length reduction similar with which induced by DSS, after regulated restraint stress. Concurrently, both bloody stool score and diarrhea score indicated the successful induction of IBD recurrence through restraint stress (Figure 3B,C).

In the stress-recurrent IBD model, the protective effects of inulin intake on intestinal inflammation was examined. The ingestion of 5% inulin significantly prevented stress-induced colon shortening (Figure 3D). Furthermore, both bloody stool score and diarrhea score were reduced compared to the cellulose group (Figure 3B,C), indicating a diminution in colonic inflammation. Fecal IgA showed a significant decrease after stress in inulin group but not in cellulose group. The IgA level before stress seemed to be elevated by inulin intake, but was not significant when compared with cellulose group. (Figure 3E). Additionally, inulin upregulated the concentration of mucin in feces (Figure 3F), this result also supported by real-time PCR analysis (Figure 3L). Concurrently, inulin treatment also decreased serum inflammatory markers, such as IL-6 and CALP (Figure 3G,H), prominent indicators of colitis. Consistent with these findings, real-time PCR analysis of colon samples showed a significant downregulation in the mRNA expression levels of inflammatory cytokines, such as *Il6* (Figure 3I) and *Il1b* (Figure 3K), while the expression of the anti-inflammatory factor *Tgfb1* (Figure 3J) increased but did not reach significance. These results underscore the beneficial role of inulin in preventing stress-induced colitis recurrence by mitigating colonic inflammation. 

### 2.4. Inulin Improved IBD Symptoms by Augmenting SCFAs and Alleviating Endoplasmic Reticulum (ER) Stress

The studies described above indicated that inulin intake can improve intestinal inflammation in stress recurrent IBD model. To examine the potential associations of improved intestinal metabolites by inulin and colonic inflammation, the short-chain fatty acids (SCFAs) concentration in cecal contents and endoplasmic reticulum stress marker expression level were tested.

**Figure 4:**
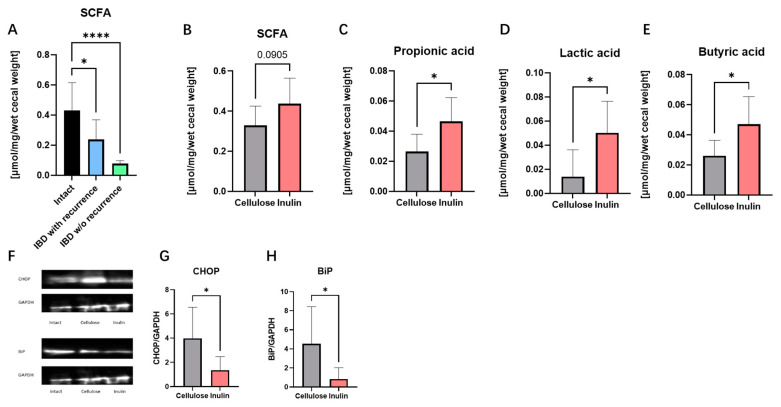
Both stress and DSS reduced SCFAs in cecal content (**A**). Inulin intake improved SCFA concentration (**B**), especially about propionic acid (**C**), lactic acid (**D**) and butyric acid (**E**). Western blot results (**F**) showed protein expression level of ER stress related gene CHOP (**G**) and BiP (**H**). Data are expressed as mean ± SEM values. N = 6–8 at each group. * *p* < 0.05, **** *p* < 0.0001, unpaired *t*-test between Inulin and Cellulose and one-way ANOVA between Intact, IBD w/o recurrence, IBD with recurrence.

The total SCFAs content was found to be downregulated in stress-recurrent IBD model (Figure 4A). After inulin intake, the total SCFA content in the cecal content tended to be upregulated compared to the cellulose group (Figure 4B). In addition, significant increases in propionic acid (Figure 4C), lactic acid (Figure 4D), and butyric acid (Figure 4E) were confirmed in the inulin group. However, acetic acid did not observe significant difference.

Protein expression levels of CHOP and BiP were measured to confirm endoplasmic reticulum stress in colon tissue (Figure 4G,H). It was known that the upregulation in the expression levels of CHOP and BiP led to the occurrence of ER stress. In this experiment, the expression levels of CHOP and BiP were significantly increased in the cellulose group. Therefore, it is thought that in the cellulose group, proteins were not folded properly due to endoplasmic reticulum stress. Based on these results this study propose that inulin inhibit intestinal inflammation through improving intestinal bacteria metabolism and reducing ER stress in intestinal epithelium.

## 3. Discussion

In this research, inulin was discovered to suppress the severity of the IBD inflammation by improving intestinal health. Both in vitro and in vivo models, inulin intake led to an enhanced anti-inflammatory effects through suppressing proinflammatory cytokine expressions and elevating IgA and other mucosal immune defenses. Furthermore, our results showed that inulin suppressed the inflammatory responses in acute stress-recurrent IBD conditions, rather than a simple anti-inflammatory prebiotic effects against pro-inflammatory factors. In this study, through the comparison of bloody stool score and diarrhea score (Figure 3B,C), stress was demonstrated to induce the recurrence of IBD. In our stress model, an increase in the concentration of corticosterone in the serum was observed. According to previous reports, prolonged exposure to stress can lead to heightened levels of corticosterone, exacerbating the physiological burden [5]. This may result in the overexpression of glucocorticoids, adrenocorticotropic hormone (ACTH), and pro-inflammatory cytokines, thereby influencing the onset of neuropsychiatric and metabolic disorders [18]. In our study, two weeks of regular stress induced an increase in the expression levels of pro-inflammatory cytokines, consisting with current research findings. These results suggested that changes in immune cell activities induced by the stress, leading to an imbalance in the production of proinflammatory cytokines, may be a possible cause for this recurrence.

Previous studies have indicated a close relationship between IBD and dysregulation of the intestinal immune system [19]. It is known that inflammatory cytokines such as TNF-α, IL-1β, IL-6, and TGF-α can disrupt intestinal barrier function, leading to bacterial invasion and excessive inflammation [20]. Currently, the treatment of IBD primarily involves immunosuppressive agents such as corticosteroids and anti-TNF agents like infliximab [21]. In this research, a significant reduction in the expression of proinflammatory cytokines in colon samples from the inulin group was observed (Figure 3I,K), with the improvement of IBD symptoms (Figure 3B–D). In addition, inulin intake upregulated the expression mRNA level of *Pigr* and IgA concentration in stool after stress (Figure 2E,G). This suggests that the anti-inflammatory effects of inulin play a crucial role in improving IBD symptoms. However, the molecular targets or mechanisms of inulin anti-inflammation function is still unclear. Current study showed that inulin can improve the permeability of blood-brain barrier to prevent lipopolysaccharide (LPS) brain penetration [22]. And our previous study has demonstrated that inulin can regulate channel forming tight junctions (claudins) circadian expression [15]. According to the results above, the relationship between inulin and intestinal barrier is anticipated to play an important role in resisting inflammation triggered by stress.

Our study indicated that, in addition to the inhibition of proinflammatory factors, inulin also induced the concentration of mucin in stool (Figure 2F). Our real-time PCR data also showed that inulin upregulated the expression mRNA of *Muc2* both in vitro and in vivo (Figure 1D and Figure 2H). Research suggests that stress can impact mucin expression, potentially leading to changes in the quantity and quality of the mucus layer [23]. Altered mucin expression may result in changes in intestinal permeability, allowing harmful substances to break the intestinal epithelium and trigger inflammatory responses [24]. In this study, an increase in the expression level of *MUC2* and an improvement in the reduced fecal mucin in stress-recurrent IBD model were observed. These results suggested that inulin may have a positive impact on the intestinal mucosa, including the augmentation of mucin expression and release.

In this experiment, a significant reduction in the concentration of SCFAs in the cecal samples of the DSS-treated only model and stress-recurrent model was observed (Figure 4A). It has been reported that a decrease in SCFAs, attributable to microbial dysbiosis, is a salient characteristic of IBD [25,26]. Our studies in stress-recurrent model revealed that inulin can upregulate the production of SCFAs in cecal content (Figure 4B), especially lactic acid, propionic acid, and butyric acid (Figure 4C–E). Previous studies suggest that inulin, as a prebiotic, can ameliorate the composition of intestinal flora by promoting the growth of probiotics [27]. In this study, the cecal content weight change also suggested that inulin may have changed the intestinal flora (Figure 1A) [16]. Simultaneously, improvement in intestinal flora can increase in the production of SCFAs in intestinal metabolites. Interestingly, a previous study also reported that SCFAs could modulate intestinal immunity by inhibiting histone deacetylase (HDAC) [28,29]. In our stress-recurrent IBD model, the concentration of SCFAs in the inulin group significantly increased, providing evidence that inulin intake can counteract the intestinal bacteria dysbiosis caused by stress. In addition, studies suggested that butyric acid, as a major kind of SCFAs, produced by microbial metabolism can upregulate mucus synthesis and the expression of MUC2 in goblet cells [30]. Therefore, butyric acid is speculated to play a key role in improving stress-recurrent IBD by inulin, but still require further research to be determined.

ER stress occurs due to an imbalance between the protein-folding load within the endoplasmic reticulum and its capacity to fold proteins correctly. The unfolded protein response (UPR) is activated to restore ER homeostasis. Prolonged or severe ER stress can lead to sustained UPR activation, contributing to cellular dysfunction. Previous research has indicated an increased expression of UPR-related genes in the intestinal tissues of colitis animal models [31,32]. In addition, NF-κB activation as a pivotal factor in intestinal inflammation has been reported that have a close association with ER stress [33,34,35,36]. In this experiment, specific markers for endoplasmic reticulum stress, including BiP and CHOP were measured. The results confirmed that the ingestion of inulin improved the increase in ER stress-specific markers BiP and CHOP in the recurrent model of IBD (Figure 4G,H). This suggests that inulin can assist in the normal expression of proteins in intestinal epithelial cells by inhibiting ER stress. This could be the other possible mechanism by which inulin mediates mucin expression in IBD.

However, in this study, significant effect of inulin on tight junctions was not observed. Simultaneously, in the stress-recurrent IBD model, a significant IgA secretion improvement by inulin intake was failed to be detected. This may be related to limitations in our model, if the stress and recovery cycles are increased, the model may have result in better simulating IBD symptoms. In conclusion, our evidence suggests that inulin can suppress inflammation severity induced by stress and reduce the risk of IBD recurrence caused by stress.

## 4. Materials and Methods

### 4.1. Cell Culture

Caco-2 cell (ATCC# HTB-37_TM_) and HT-29 cell (ATCC # HTB-38_TM_) was purchased from European Collection of Authenticated Cell Cultures (ECACC; Salisbury, UK). Cells were cultured in Dulbecco’s modified Eagle medium (DMEM; WAKO, Tokyo, Japan) supplemented with 10% (*v*/*v*) fetal bovine serum (FBS; HyClone, Cytiva (Marlborough, MA, USA) and 1% (*v*/*v*) Penicillin-Streptomycin-Glutamine (Gibco, Thermo Fisher Scientific, Tokyo, Japan). Cell cultures were maintained at 37 °C and 5% CO_2_ incubator. The cells were passaged once a week and replaced media once every three days. In this study, cells were passaged at least three times before use.

### 4.2. Inulin and Cellulose Digesta Preparation

Female SPF:ICR mice aged 8 weeks were used to prepare inulin or cellulose digesta. After 1 week fed with American Institute of Nutrition (AIN)-93M which mixed 5% inulin or 5% cellulose, Mice were euthanized and the cecal contents were collected. Collected cecal content was diluted using DMEM (10% *w*/*v* as final concentration). Then, the suspension was sonicated in a water bath (3 times of 30 s each) and centrifuged (1000× *g*, 5 min). Supernatant was sterile-filtered with 0.22-μm pore filters and was stored at −80 °C. This digesta preparation was described in previous study [37].

### 4.3. Total Cell RNA Extraction and Real-Time PCR

Caco-2 cells (2.4 × 10^4^ cells/well) and HT-29 cells (0.6 × 10^4^ cells/well) were plated in 24 wells plate as a co-culture system for RNA extraction. Co-culture system was in combination with inulin or cellulose digesta (1% *w*/*v* as final concentration) for 2 h. For cytokine production via ELISA, the supernatant was collected. Total RNA was isolated from cells using QIAzol Lysis Reagent (QIAGEN, Hilden, Germany). Then collected RNA was reverse transcribed with ReverTra Ace qPCR RT Master Mix with gDNA Remover kit (Toyobo, Osaka, Japan). The 1.2 µL transcribed cDNA (1 ng/μL after dilution) was mixed with 9.0 µL qPCR reaction mixtures containing 300 nM gene specific primers (Integrated DNA Technologies, Singapore) and Thunderbird SYBR qPCR mix (Toyobo, Osaka, Japan). Primers used in this study are listed in Table 1. Copy number of gene expression was analyzed using StepOne Real-Time PCR System (RT-PCR; Applied Biosystems, Waltham, MA, USA) and copy number in expression was calculated using standard curve method using *GAPDH* as a reference gene and normalized with untreated control.

### 4.4. TNF-α ELISA

TNF-α levels were quantified using a Human TNF-α DuoSet ELISA Kit (R&D Systems, Minneapolis, MN, USA) according to the manufacturer’s protocols.

### 4.5. Animals

Female Kwl:ICR mice, were obtained from Tokyo Laboratory Animal Science (Tokyo, Japan). The mice were maintained in a conventional room maintained at 22 ± 2 °C and 60 ± 5% humidity under a 12-h light (08:00–20:00)/dark cycle (20:00–08:00). Zeitgeber time 0 (ZT0) was designated as lights-on time and ZT12 as lights-off time. The mice were provided with an AIN-93M diet and water. Experiments were performed in a non-blinded condition. This study was approved by the Committee for Animal Experimentation at Waseda University [approval number: 2019-A61] as well as at Tokyo Institute of Technology [approval number: 2022006]. Animals were treated in accordance with the committee’s guidelines.

### 4.6. Restraint Stress

In this study, mice were subjected to restraint stress by placing them in 50 mL centrifuge tubes (BD Falcon, Becton, Dickinson and Company, Tokyo, Japan). Restraint stress was applied for 2 h. 

### 4.7. Measurements of Bloody Stool Score and Diarrhea Score

In this study, IBD model mice were built by giving DSS 5% water for 13 days. Bloody stool and diarrhea score was used to evaluate IBD model mice. The appearance of blood in the stool was given a score from 0 to 3, defined as follows: 0 = no blood, 1 = blood observed when feces is crushed with wipes, 2 = blood trace in the stool clearly visible, 3 = gross rectal bleeding. The diarrhea was given a score from 0 to 3, defined as follows: 0 = well-formed stool pellet, 1 = feces stick (even a part) on the cage and bedsheet, 2 = semi-formed stool, 3 = liquid stool that adhere to anal region. When the score was >0, it was considered that the mice was exhibiting IBD symptoms, and the higher the score, the higher the severity.

### 4.8. Stool Collection

In this study, mouse stool were used to measure IgA and mucin. Since fecal IgA may be degraded by endogenous proteases after defecation, fresh stool was collected into a protease inhibitor (Sigma-Aldrich, Tokyo, Japan), complete^TM^ Protease Inhibitor Cocktail) containing micro-centrifuge tubes, and incubated on ice. Then, it was homogenized at 12,000 rpm for 15 min. The fecal supernatant was collected and stored at −80 °C.

### 4.9. IgA ELISA

Fecal IgA levels were quantified using an IgA Mouse Uncoated ELISA Kit (Thermo Fisher Scientific, Tokyo, Japan), according to the manufacturer’s protocols.

### 4.10. Mucin ELISA

Fecal Mucin levels were quantified using a Fecal Mucin Assay Kit (Cosmo Bio, Tokyo, Japan), according to the manufacturer’s protocols.

### 4.11. Blood Collection

Blood was collected from mice by anesthesia with isoflurane (FUJIFILM Wako Pure Chemical Corporation, Osaka, Japan). Blood was collected from the cheek, using animal lancets. Subsequently, the blood was placed into a 1.5 mL tube and homogenized, followed by the extraction of serum.

### 4.12. Corticosterone ELISA

Serum corticosterone levels were quantified using the AssayMax Corticosterone ELISA Kit (Assay Pro, MO, USA) according to the manufacturer’s protocol.

### 4.13. IL-6 ELISA

Serum IL-6 levels were quantified using the Mouse IL-6 DuoSet ELISA Kit (R&D Systems, Minneapolis, MN, USA) according to the manufacturer’s protocol.

### 4.14. CALP ELISA

Serum CALP levels were quantified using the Mouse calprotectin (CALP) ELISA Kit (CUSABIO, Houston, TX, USA) according to the manufacturer’s protocol.

### 4.15. Tissue Collection

Mice were euthanized and the colon was collected. Tissues were placed into protease inhibitor-containing microcentrifuge tubes and kept on ice. Samples were homogenized using a micro homogenizer and then centrifuged at 3000× *g* for 20 min at 4 °C. The supernatants were stored at −80 °C until the analyses were performed.

### 4.16. Isolation of Mice Total RNA and Real-Time RT-PCR

Total RNA was extracted from tissues, using phenol. Aliquots of 50 ng of total RNA were reverse-transcribed and amplified using a One-Step SYBR reverse transcription PCR (RT-PCR) kit (TaKaRa Bio, Shiga, Japan) in Piko Real (Thermo Fisher Scientific, Tokyo, Japan). Primer pairs were designed using OligoAnalyzer software (Integrated DNA Technologies, Singapore) and the primers used in this study are listed in Table 1. The relative level of target gene was calculated using 2^−ΔΔCT^ method using *18srrna* as a reference gene and normalized with untreated control.

### 4.17. Western Blot

Colon tissues were homogenized in radioimmunoprecipitation assay (RIPA) buffer (Sigma-Aldrich, Tokyo, Japan) and then separated by centrifugation at 14,000× *g* at 4 °C for 60 min. Colon lysates were adjusted to 50 μg using a spectrophotometer (NanoVue, GE Healthcare). Total proteins were electrophoresed on SDS-polyacrylamide gels and transferred to an Immobilon polyvinylidene difluoride (PVDF) membrane. After blocking with TBST solution (25 mM Tris, 135 mM NaCl, 2.5 mM KCl, 0.1% Tween 20, pH 7.4) for 1 h at room temperature, the membrane was then incubated with respective antibodies at 4 °C overnight. Then, the blots were washed and incubated with the secondary antibody for 1 h. After 4 times TBST buffer washing, Blots were developed using the ECL immunoblotting detection system (Amersham Biosciences, Piscataway, NJ, USA) and imaged using the ChemiDoc™ Touch Imaging System (Bio-Rad, Tokyo, Japan). Anti-bodies for CHOP (L63F7) and BiP (C50B12) were contained in ER Stress Antibody Sampler Kit #9956 which purchased from Cell Signaling Technologies (Danvers, MA, USA).

### 4.18. Gas Chromatography Analysis

Cecal contents (30 mg) were added with 50 μL of 1 mol sulfuric acid. 600 μL ether and chloroform solution (2:1) was added to each sample, and vortexed. Cecal contents were centrifuge at 14,000 rpm for 30 s at room temperature. Supernatant was added with 100 μL of derivatization reagent and then heat at 60 °C for 30 min using a heat block. After cooling on ice for 10 min, samples were centrifuged again at 14,000 rpm for 30 s at room temperature. The supernatant was performed quantitative analysis using gas chromatography (Shimadzu, Kyoto, Japan).

### 4.19. Statistical Analysis

All data were represented graphically as mean values with standard errors. Statistical analysis was performed using GraphPad Prism (version 6.03–9.30; GraphPad Software, Boston, MA, USA). The normality of data distribution or biased variation was assessed using the Kolmogorov-Smirnov test or the F value test or Bartlett’s test. Parametric analysis was conducted by one-way, one-way repeated or two-way ANOVA with a Tukey test and Student’s *t*-test. Non-parametric analysis was performed by a Kruskal-Wallis or Friedman test with Dunn and Mann-Whitney tests. Statistical significance was set at *p* < 0.05, and *p* < 0.15 data are shown.

## 5. Conclusions

In conclusion, all these results showed that inulin intake suppressed intestinal immunity to be decreased by restraint stress. Indeed, inulin alleviates IBD-like symptoms based on a stress-recurrent IBD model, such as bloody stools, diarrhea, and colon shortening. This study revealed that inulin improves intestinal immune function in stress-recurrent IBD model mice by maintaining mucin function and regulating the expression of inflammatory cytokines. The functions of inulin on intestinal microbial metabolites and ER stress may be the potential mechanisms. These findings suggest that inulin can be useful for future dietary therapy for IBD and improve patients’ quality of life. Additionally, the rate of fermentation of inulin varies depending on its degree of polymerization. Therefore, future studies to investigate an appropriate timing of inulin intake and the ratio of inulin intake are expected to contribute to the development of IBD treatment.

## Figures and Tables

**Table 1:** Primer sequences.

Gene Name	Forward (5′-3′)	Reverse (5′-3′)
(Human)		
*GAPDH*	CAGCCTCAGTACAGCAATCAAC	TAGGGGTCATAGGAGTCATTGG
*CXCL8/IL8*	GGCAGCCTTCCTGATTTGTG	GCTTTACAATAATTTCTGTGTTGG
*TNFA*	GCTTTGATCCCTGACATCTGG	GGAAACATCTGGAGAGAGGAAG
*MUC2*	GAAGTGAAGAGCAAGATGGTG	GGAGGAATAAACTGGAGAACC
(Mice)		
*18srrna*	CGAAAGCATTTGCCAAGAAT	GCGGGTCATGGGAATAAC
*Pigr*	AGTAACCGAGGCCTGTCCT	GTCACTCGGCAACTCAGGA
*Claudin3*	ACCCACCAAGATCCTCTATT	TAGTCCTTGCGGTCGTAG
*Claudin4*	GGGCAAGAGGGAAATG	TAGGGCTTGTCGTTGCTA
*Occludin*	CACACTTGCTTGGGACAG	GGGTCTGTATATCCGCCATA
*Zo1*	GAAACTGATGCTGTGGATAGA	GGAATGTATGTGGAGA
*Muc2*	CTCATCATGGACAGCCTATTC	CACTGGCACACTCCATATTAG

## Data Availability

The original contributions presented in the study are included in the article, further inquiries can be directed to the corresponding author.
